# Imaging Markers of Subcortical Vascular Dementia in Patients With Multiple-Lobar Cerebral Microbleeds

**DOI:** 10.3389/fneur.2021.747536

**Published:** 2021-11-12

**Authors:** Chia-Yen Lin, Song-Ru Jhan, Wei-Ju Lee, Po-Lin Chen, Jun-Peng Chen, Hung-Chieh Chen, Ting-Bin Chen

**Affiliations:** ^1^Department of Neurology, Neurological Institute, Taichung Veterans General Hospital, Taichung, Taiwan; ^2^Division of Neuroradiology, Department of Radiology, Taichung Veterans General Hospital, Taichung, Taiwan; ^3^Dementia Center, Taichung Veterans General Hospital, Taichung, Taiwan; ^4^School of Medicine, National Yang-Ming Chiao Tung University, Taipei, Taiwan; ^5^Biostatistics Task Force of Taichung Veterans General Hospital, Taichung, Taiwan; ^6^Department of Applied Cosmetology, Hungkuang University, Taichung, Taiwan

**Keywords:** hypertensive angiopathy, amyloid angiopathy, lacune, microbleed, white matter hyperintensities

## Abstract

**Background and Purpose:** Small vessel disease (SVD) imaging markers are related to ischemic and hemorrhage stroke and to cognitive dysfunction. This study aimed to clarify the relationship between SVD imaging markers and subcortical vascular dementia in severe SVD burden.

**Methods:** A total of 57 subjects with multiple lobar cerebral microbleeds (CMBs) and four established SVD imaging markers were enrolled from the dementia and stroke registries of a single center. Visual rating scales that are used to semi-quantify SVD imaging changes were analyzed individually and compositely to make correlations with cognitive domains and subcortical vascular dementia.

**Results:** Dementia group had higher subcortical and total white matter hyperintensities (WMHs) and SVD composite scores than non-dementia group. Individual imaging markers correlated differently with one another and had distinct cognitive correlations. After adjusting for demographic factors, multivariate logistic regression indicated associations of subcortical WMHs (odds ratio [OR] 2.03, CI 1.24–3.32), total WMHs (OR 1.43, CI 1.09–1.89), lacunes (OR 1.18, CI 1.02–1.35), cerebral amyloid angiopathy-SVD scores (OR 2.33, CI 1.01–5.40), C_1_ scores (imaging composite scores of CMB and WMH) (OR 1.41, CI 1.09–1.83), and C_2_ scores (imaging composite scores of CMB, WMH, perivascular space, and lacune) (OR 1.38, CI 1.08–1.76) with dementia.

**Conclusions:** SVD imaging markers might have differing associations with cognitive domains and dementia. They may provide valuable complementary information in support of personalized treatment planning against cognitive impairment, particularly in patients with a heavy SVD load.

## Introduction

Small vessel disease (SVD) is characterized by a variety of cerebral parenchymal lesions that can be detected by neuroimaging, including cerebral microbleeds (CMBs), white matter hyperintensities (WMHs), lacunes, and enlargements of the perivascular space (PVSEs) (1). These SVD-related MRI markers have been associated with ischemic and hemorrhagic stroke and dementia (1), and these features have also been linked to vascular risk factors, cognitive dysfunction, depression, and epilepsy (2, 3). These markers often co-occur, together constituting a composite SVD burden. Several studies using composite scoring scales of the salient SVD-related MRI signatures have demonstrated a negative impact of global SVD burden on cognition, functional ability, gait, and balance (4–8).

Cerebral microbleeds increase the risk of spontaneous intracerebral hemorrhage (ICH), ischemic stroke, cognitive impairment, and gait disturbance (9, 10). Two main sporadic forms of SVD present with CMBs: hypertensive arteriopathy (HA) and cerebral amyloid angiopathy (CAA). The former affects the small perforating end arteries of deep gray nuclei and deep WM. The latter is characterized by progressive amyloid-beta deposition in the tunica media and adventitia of small cortical vessels overlying the leptomeninges and gray-WM junction. The topographical CMB distribution could be utilized to identify the underlying SVD pathogenesis. Deep and infratentorial (i.e., nonlobar) CMBs are indicative of HA, whereas strictly lobar CMBs suggest CAA (11). Multiple lobar CMBs, like strictly lobar CMBs, could suggest CAA pathology (12). A mix of lobar with nonlobar CMBs can occur in HA, but a HA-CAA synergism could also be responsible for CMB pathogenesis, which implies an SVD spectrum (10, 11, 13).

White matter hyperintensities are predictive of stroke risk and have been associated with declining global cognition, executive function, and processing speed, as well as with dementia, depression, and death due to cardiovascular causes (14). Periventricular WMHs and deep WMHs have distinct pathophysiologies and clinical significances (15). Major risk factors for WMH development are advanced age, hypertension, and cardiovascular disease. The underlying pathology of WMHs in subcortical vascular dementia involves demyelination and axon loss due to SVD ischemia (16).

The PVS is the fluid-containing space surrounding blood vessels, as they course from the subarachnoid space into the brain (17). PVSEs may result from obstructed drainage (17). Basal ganglia (BG) PVSEs have been associated with advanced age, hypertension, and stroke, whereas centrum semiovale (CS) PVSEs have been associated with CAA or a mixed hypertensive/CAA pathophysiology (18, 19).

Although SVD changes are commonly seen in routine MRIs, little is known about their cognitive contributions in patients with multiple lobar CMBs. We used a validated CAA-SVD scoring system, showing close association with the severity of CAA-related vasculopathic changes at autopsy, to represent the underlying CAA load in the brain in our cohort (4, 20). Additionally, we developed two comparable ordinal scores based on the four established MRI markers of SVD to compile the overall brain burden. The primary aim of this work was to investigate associations of individual regional imaging markers and composite scores with cognitive performance and dementia risk in patients with multiple lobar CMBs and coexisting all four MRI features of SVD.

## Methods

### Subjects

We identified patients with dementia or acute stroke from the dementia care database and stroke registry at Taichung Veterans General Hospital from January 2017 to March 2020. The institutional review board and the ethics committee of Taichung Veterans General Hospital approved the data collection protocol. Informed consent was waived by ethics committee of Taichung Veterans General Hospital.

We first considered all subjects over 55 years old diagnosed with subcortical vascular dementia, acute symptomatic lacunar stroke, or acute symptomatic spontaneous lobar ICH. Those with all four features of SVD (i.e., CMBs, WMHs, PVSEs, and lacunes) and multiple lobar CMBs visible on MRI were enrolled. Multiple (≥ 2) lobar was defined as involving more than one (≥ 2) frontal, parietal, temporal, or occipital lobes of right or left hemisphere, with or without CMBs in a deep or infratentorial region. The exclusion criteria were: age <55 years; diagnosis with an uncertain dementia; acute ischemic stroke due to causes other than small-vessel occlusion; ICH due to head trauma; hemorrhagic transformation of a stroke; arteriovenous malformation; hemorrhagic tumor; recipient of anticoagulant therapy; and vasculitis.

Two well-trained licensed nurse case managers conducted registration management, including collection of a brief history, examination findings, age, sex, body mass index, medical comorbidities (including cardiovascular and metabolic diseases), and cigarette and alcohol usage. The Charlson comorbidity index (CCI), which contains 19 weighted comorbidities, was also gathered to determine the overall systemic health of each individual (21). Cognitive performance in the subjects with cognitive decline was assessed by neuropsychologists using 12-item word recall test (total correct trials 1–3, and 15-min delayed free recall) (22, 23), estimated Mini-Mental State Examination (MMSE) converted from Cognitive Abilities Screening Instrument (24, 25), Montreal Cognitive Assessment (MoCA) (26), and Clinical Dementia Rating scale (27). Cognitive performance was documented as well as the contents of diagnostic workups, dementia disorder type, pharmacological and non-pharmacological treatments, and support for the patient from the county.

### Clinical Diagnosis and Grouping

Medical records and registry data were reviewed in detail. Subjects were dichotomized into dementia group and non-dementia group. Dementia diagnoses were made in accordance with standard criteria based on a clinical interview, functional assessments, neurological examinations, neuropsychological screening, MRI, and laboratory studies (28). Probable Alzheimer's disease was diagnosed when subjects meet the clinical criteria proposed by the National Institute of Neurological and Communicative Disorders and Stroke/Alzheimer's Disease and Related Disorders Association (29) and the National Institute on Aging/Alzheimer's Association workgroup (30). Diagnoses of vascular dementia, frontotemporal dementia, and dementia with Lewy bodies were made according to their respective criteria (31–34).

Acute stroke was confirmed by board-certified neurologists and/or neurosurgeons based on clinical symptoms, neurological signs, and brain MRI findings. Ischemic stroke subtypes were classified into five etiological categories in accordance with the Trial of ORG 10172 in Acute Stroke Treatment (TOAST) rubric (35).

### Brain MRI

All subjects were scanned in a 1.5-T MRI scanner (MAGNETOM Aera, Siemens Healthcare, Erlangen, Germany). MRI scanning was done within 3 months after cognitive assessment in the patients with dementia and in the setting of acute clinical event in the patients with acute stroke. MRI was performed according to a standardized protocol inclusive of the following sequences: axial spin echo (SE) T1-weighted images (T1WI) [repetition time (TR)/echo time (TE) = 550/8.9 ms, Field-of-View (FOV) = 230^*^230 mm^2^, matrix size = 320^*^256, slice thickness = 6 mm], axial turbo-SE (TSE) T2-weighted images (T2WI) (TR/TE = 3,500/103 ms, FOV = 230^*^208 mm^2^, matrix size = 512^*^384, slice thickness = 6 mm), axial fluid-attenuated inversion recovery (FLAIR) (TR/TE/TI = 9,000/86/2,500 ms, FOV = 230^*^201 mm^2^, matrix size = 320^*^240, slice thickness = 6 mm), axial diffusion weighted images (DWI) (TR/TE = 6,300/89 ms, FOV = 230^*^230 mm^2^, matrix size = 192^*^192, slice thickness = 6 mm), oblique coronal TSE T2-weighted images (T2WI) for temporal lobe (TR/TE = 4,000/83 ms, FOV = 180^*^180 mm^2^, matrix size = 384^*^307, slice thickness = 3 mm), axial 3D time-of-flight magnetic resonance angiography (3D-TOF-MRA) (TR/TE = 24/7 ms, FOV = 180^*^180 mm^2^, matrix size = 256^*^218, flip angle = 25°, slice thickness = 0.5 mm, slabs = 4 slices per slab = 52), and axial susceptibility-weighted MRI (SWI) (TR/TE = 49/40 ms, FOV = 230^*^186 mm^2^, flip angle = 15°, matrix size = 288^*^230, slice thickness = 2 mm). The total scan time is about 35 min.

### Visual Rating

Two independent experienced neuroradiologists rated the MRI data. CMBs, WMHs, and PVSEs were defined according to the Standards for Reporting Vascular changes on Neuroimaging (STRIVE) consensus (36). CMBs were recognized as ≤ 10 mm homogeneous-rounded hypointense lesions on SWI. CMBs were counted in lobar, deep, and infratentorial regions based on the Microbleed Anatomical Rating Scale (MARS) (37). Ill-defined hyperintensities ≥ 5 mm on T2WI and FLAIR images were recognized as WMHs. WMHs detected in five brain regions (frontal lobe, parietal-occipital lobe, temporal lobe, BG, and infratentorial region) in the hemisphere of more severe WMH were graded according to the Age-Related White Matter Change (ARWMC) scale (range, 0–3) (38). The ARWMC scale scores for each lobe aggregated by hemisphere were designated as subcortical ARWMC scale. All ARWMC scores in the five brain regions were combined to calculate total ARWMC score that reflected WMH severity of the entire hemisphere. The ARWMC scale has been shown to correlate with WMH volume (39, 40). Periventricular and deep WMHs were graded according to a modified Fazekas scale (range, 0–3) (41). The PVS regions were considered enlarged when spaces measuring > 3 mm were visible with signal intensity similar to cerebrospinal fluid on T2WI (36). The PVSEs were counted and rated semi-quantitatively in the BG and CS as follows: 0, no PVSEs; 1, 1–10; 2, 11–20; 3, 21–40; and 4, >40 PVSEs (42). We counted ICHs and lacunes visible on FLAIR and SWI sequences. Cortical superficial siderosis (cSS) was identified as a curvilinear hypointensity following the gyral surface on SWI. There was high inter-rater reliability, and the intra-class correlation coefficients between the raters for MARS total score and ARWMC total score were between 0.98 and 0.99.

### SVD Composite Scoring

We adopted a validated CAA-SVD scoring reflexive of total CAA pathological burden in the brain (4, 20). Briefly, each marker category was scored on a 0–6 scale. Lobar CMBs were scored 1 point if there were 2–4 CMBs or 2 points if there were ≥ 5. Focal cSS was scored 1 and disseminated cSS was scored 2 points. One point (each) was scored for ≥ 20 CS PVSEs and WMHs with periventricular Fazekas score ≥ 3 or a deep Fazekas score ≥ 2. We generated a composite score 1 (C_1_) comprising the total MARS grade (1 point for 2–4 CMBs; 2 points for ≥ 5 CMBs) and the total ARWMC score. A composite score 2 (C_2_) consisted of C1, BG PVSE, and lacune amount. One point (each) was scored for ≥ 20 BG PVSEs or ≥ 5 lacunes. C_2_ score was equivalent to the global cerebral SVD score developed to represent total SVD burden of both HA and CAA pathologies (20).

### Statistical Analysis

Analyses were performed in Statistical Package for the Social Sciences (SPSS) version 22.0 for Windows (SPSS Inc., Chicago, IL). Two-tailed *p* < 0.05 were considered significant. Differences in continuous/categorical variables between patients with *vs*. without subcortical vascular dementia were detected with Mann-Whitney *U*-test/Chi-square tests. We generated neurocognitive numeric composite z-scores by calculating individual z-scores for each test and then averaging them across the cognitive test set. The constituents of the composite z-scores were as follows: attention [Serial 7s on the MMSE and sustained attention task and forward and backward digit span on the MoCA], short-term memory (registration) [total number of items remembered over three trials on the 12-item memory test, immediate memory task on the MMSE], short-term memory (recall) [15-min delayed recall on the 12-item memory test, delayed memory recall task on the MMSE and MoCA]; orientation [orientation tests on the MMSE and MoCA]; language [confrontation naming task, repetition of two syntactically complex sentences, writing, and fluency task on the MMSE and MoCA]; visuoexecutive function [pentagon test on the MMSE and clock-drawing task and a three-dimensional cube copy on the MoCA]. Spearman's rank coefficients were calculated to determine the correlation of imaging variables with each other and constituent cognitive domains. To investigate the association of imaging variables with dementia diagnosis, we developed a logistic regression model after adjustment for age, sex, and CCI. Adjusted odds ratio (OR) values are reported with 95% CIs.

## Results

### Demographic and Imaging Characteristics of the Entire Cohort

Fifty-seven patients with multiple lobar CMBs were divided into dementia group (*N* = 12) and non-dementia group (*N* = 45). The two groups differed significantly with respect to subcortical and total ARWMC scale score, CAA-SVD score, C_1_ score, and C_2_ score (Tables 1, 2).

**Table 1:** Demographic and clinical characteristics of this cohort, *N* = 57.

**Characteristic**	**Dementia** **(*N =* 12)**	**Non-dementia** **(*N =* 45)**	***P***
Sex, female, *N* (%)	4 (33.3%)	13 (28.9%)	0.737
Age, years	74.5 (71.5–82.3)	72.0 (63.5–82.5)	0.337
Smoking, *N* (%)	6 (50.0%)	16 (35.6%)	0.506
Hypertension, *N* (%)	10 (83.3%)	41 (91.1%)	0.596
Diabetes mellitus, *N* (%)	3 (25.0%)	14 (31.1%)	1.000
Hyperlipidemia, *N* (%)	4 (33.3%)	24 (53.3%)	0.365
Peripheral or cardiac vasculopathy^†^, *N* (%)	2 (16.7%)	11 (24.4%)	0.713
Atrial fibrillation, *N* (%)	1 (8.3%)	1 (2.2%)	0.380
CCI	2.5 (1.3–3.8)	2.0 (2.0–3.0)	0.928
Antiplatelet use, *N* (%)	7 (58.3%)	24 (53.3%)	1.000
Anticoagulant use, *N* (%)	1 (8.3%)	1 (2.2%)	0.380

*ICH, intracerebral hemorrhage; CCI, Charlson comorbidity index*.

*Continuous variables, presented as median values and interquartile ranges (IQRs), were analyzed with the Mann-Whitney U-test; categorical variables, presented as number of patients with percentage, were examined with the Chi-square test*.

† *Includes: peripheral artery disease, coronary artery disease, and carotid artery disease*.

**Table 2:** Between-group comparison of imaging variables, *N* = 57.

**Variable**	**Dementia**	**Non-dementia**	***P***
**MARS**			
Infratentorial	4.0 (0.0–10.5)	1.0 (0.0–8.0)	0.545
Deep	4.5 (0.25–32.0)	2.5 (0.0–13.75)	0.423
Lobar	30.50 (4.3–55.0)	5.5 (2.0–22.25)	0.105
Total	32.5 (7.5–88.0)	11.5 (3.0–39.75)	0.119
**Cortical superficial siderosis**	6 (50.0%)	10 (22.2%)	0.076
**ARWMC**			
Infratentorial	0.0 (0.0–0.0)	0.0 (0.0–0.5)	0.572
Basal ganglia	2.0 (1.0–2.0)	2.0 (1.0–2.0)	0.299
Subcortical	6.0 (5.0–7.0)	4.0 (3.0–5.5)	0.003^*^
Total	9.5 (8.0–11.75)	8.0 (4.0–10.0)	0.015^*^
**PVSE**			
Centrum semiovale	3.0 (2.0–3.75)	3.0 (2.5–4.0)	0.383
Basal ganglia	3.0 (2.0–3.0)	3.0 (2.0–3.0)	0.644
**Lesion quantity**			
ICH	2.0 (0.25–3.5)	2.0 (0–4.0)	0.795
Lacune	8.0 (3.25–11.75)	3.0 (2.0–6.5)	0.05
**Lesion burden score**			
CAA-SVD score	4.0 (3.25–5.0)	3.0 (2.0–4.0)	0.027^*^
C_1_^†^	11.5 (9.25–13.75)	9.0 (6.0–11.5)	0.008^**^
C_2_^‡^	13.0 (11.0–14.75)	10.0 (7.0–13.0)	0.007^**^

*MARS, Microbleed anatomical rating scale; ARWMC, Age-related white matter change; PVSE, enlargement of the perivascular space; CAA, cerebral amyloid angiopathy; SVD, small vessel disease*.

*Continuous variables, presented as median (interquartile ranges), subjected to Mann-Whitney U-test; Categorical variables, presented as the number of patients (percentage) subjected Chi-square test*;

* *p < 0.05*,

** *p < 0.01*.

† *Sum of total MARS score and total ARWMC scale*.

‡ *Sum of total MARS score, total ARWMC scale, BG PVSE (≥20), and lacune amount (≥5)*.

### Correlations of Imaging Variables Within Variable Categories of the Entire Cohort

Most MARS and ARWMC scale items showed fair correlation with each other and moderate-to-strong correlation with CAA-SVD score, C_1_ score, and C_2_ score (Table 3). PVSE did not correlate with any other items except C_2_ score. ICH amount fairly correlated with CAA-SVD score and C_1_ score, while lacune burden fairly correlated with C_2_ score (Table 3).

**Table 3:** Correlations of individual imaging variables of the cohort, *N* = 57.

		**MARS**				**ARWMC**				**PVSE**		**Lesion quantity**		**Lesion burden score**		
**Factors**		**Infratentorial**	**Deep**	**Lobar**	**Total**	**Infratentorial**	**BG**	**Subcortical**	**Total**	**CS**	**BG**	**ICH**	**Lacune**	**CAA-SVD**	**C_**1**_**	**C_**2**_**
MARS	Infratentorial	–														
	Deep	0.734^**^	–													
	Lobar	0.659^**^	0.603^**^	–												
ARWMC	Infratentorial	0.306^*^	0.298^*^	0.228	0.259	–										
	BG	0.334^*^	0.205	0.288^*^	0.338^*^	−0.008	–									
	Subcortical	0.423^**^	0.247	0.492^**^	0.484^**^	0.272^*^	0.392^**^	–								
PVSE	CS	0.059	−0.025	−0.083	−0.059	0.058	−0.113	−0.179	−0.164	–						
	BG	−0.060	0.010	−0.079	−0.101	−0.106	0.135	−0.034	0.011	0.224	–					
Lesion quantity	ICH	0.369^**^	0.373^**^	0.162	0.287^*^	0.205	0.110	0.225	0.250	0.112	−0.159	–				
	Lacune	0.283^*^	0.374^**^	0.247	0.295^*^	0.148	0.258	0.244	0.302^*^	−0.034	−0.166	−0.026	–			
Lesion burden score	CAA-SVD	0.541^**^	0.537^**^	0.773^**^	0.755^**^	0.183	0.307^*^	0.507^**^	0.558^**^	0.081	0.125	0.305^*^	0.231	–		
	C_1_	0.723^**^	0.557^**^	0.796^**^	0.819^**^	0.370^**^	0.513^**^	0.787^**^	0.849^**^	−0.167	−0.036	0.284^*^	0.238	0.684^**^	–	
	C_2_	0.625^**^	0.566^**^	0.574^**^	0.622^**^	0.346^**^	0.443^**^	0.508^**^	0.602^**^	0.372^**^	0.395^**^	0.199	0.425^**^	0.619^**^	0.682^**^	–

*ARWMC, Age-related white matter change; BG, basal ganglia; CS, centrum semiovale; ICH, intracerebral hemorrhage; MARS, Microbleed anatomical rating scale; PVSE, enlargement of the perivascular space; CAA, cerebral amyloid angiopathy; SVD, small vessel disease*.

*Spearman's rank correlation coefficient was calculated to explore correlations between variables. Dark gray block: moderate-to-strong correlation, 0.6 ≤ |r_s_|. Light gray block: fair correlation, 0.3 ≤ |r_s_| < 0.6*.

* *p < 0.05*,

** *p < 0.01*.

### Correlations of Imaging Variables With Cognitive Measures of the Dementia Group

Infratentorial and deep MARS scales moderately-to-strongly negatively correlated with short-term memory (registration and recall) and visuoexecutive function, lobar MARS scale fairly with language, and total MARS scale moderately with language and visuoexecutive function (Table 4). BG ARWMC scale moderately-to-strongly negatively correlated with global cognition (MoCA) and language, while subcortical and total ARWMC scales moderately-to-strongly negatively correlated with performance in global function (MMSE and MoCA), short-term memory (recall), orientation, and language (Table 4). Regarding the composite scores, C_1_ score outperformed CAA-SVD score and C_2_ score in the cognitive correlation (Table 4). C_1_ score moderately-to-strongly negatively correlated with global function, short-term memory (recall), orientation, language, and visuoexecutive function (Table 4).

**Table 4:** Correlations of individual imaging variables with neurocognitive domains of the dementia group, *N* = 12.

		**MMSE**	**MoCA**	**Attention**	**STM (registration)**	**STM (recall)**	**Orientation**	**Language**	**Visuoexecutive function**
MARS	Infratentorial	−0.493	−0.540	−0.239	−0.582^*^	−0.642^*^	−0.440	−0.486	−0.731^**^
	Deep	−0.376	−0.271	−0.026	−0.801^**^	−0.524	−0.400	−0.278	−0.627^*^
	Lobar	−0.410	−0.449	−0.300	−0.289	−0.417	−0.416	−0.583^*^	−0.480
	Total	−0.466	−0.561	−0.321	−0.423	−0.528	−0.463	−0.650^*^	−0.661^*^
ARWMC	Infratentorial	−0.033	0.001	0.002	0.098	−0.299	−0.066	0.131	0.073
	Basal ganglia	−0.370	−0.601^*^	−0.423	−0.181	−0.435	−0.278	−0.664^*^	−0.438
	Subcortical	−0.675^*^	−0.665^*^	−0.574	−0.022	−0.677^*^	−0.621^*^	−0.648^*^	−0.541
	Total	−0.674^*^	−0.730^**^	−0.614^*^	0.002	−0.716^**^	−0.619^*^	−0.692^*^	−0.511
PVSE	CS	0.329	0.080	−0.026	0.319	0.390	0.427	0.095	0.366
	BG	−0.131	−0.326	−0.231	0.246	−0.263	−0.074	−0.285	−0.052
Lesion quantity	ICH	−0.231	0.200	0.094	−0.695^*^	−0.172	−0.216	0.265	−0.328
	Lacune	0.002	0.056	0.307	−0.452	−0.365	−0.023	0.179	−0.079
Lesion burden score	CAA–SVD	−0.133	−0.303	−0.236	0.163	−0.192	−0.116	−0.414	−0.093
	C1	−0.675^*^	−0.744^**^	−0.563	−0.190	−0.733^**^	−0.660^*^	−0.753^**^	−0.606^*^
	C2	−0.336	−0.575	−0.351	−0.134	−0.600^*^	−0.233	−0.502	−0.315

*MMSE, Mini–Mental State Examination; MoCA, Montreal Cognitive Assessment; STM, short–term memory; ARWMC, Age–related white matter change; MARS, Microbleed anatomical rating scale; PVSE, enlargement of the perivascular space; CS, centrum semiovale; BG, basal ganglia; ICH, intracerebral hemorrhage; CAA, cerebral amyloid angiopathy; SVD, small vessel disease*.

*Spearman's rank correlation coefficient was calculated to explore correlations between variables. Dark gray block: moderate-to-strong correlation, 0.6 ≤ |r_s_|. Light gray block: fair correlation, 0.3 ≤ |r_s_| <0.6*.

* *p < 0.05*,

** *p < 0.01*.

### Associations of Imaging Variables With Dementia Diagnosis

A logistic regression controlled for age, sex, and CCI (Table 5) revealed positive associations of subcortical ARWMC (OR 2.03; 95% CI, 1.24–3.32, *p* = 0.005), total ARWMC (OR 1.43; 95% CI, 1.09–1.89, *p* = 0.011), lacune number (OR 1.18; 95% CI, 1.02–1.35, *p* = 0.023), CAA-SVD score (OR 2.33; 95% CI, 1.01–5.40, *p* = 0.047), C_1_ (OR 1.41; 95% CI, 1.09–1.83, *p* = 0.009), and C_2_ (OR 1.38; 95% CI 1.08–1.76, *p* = 0.010) with subcortical vascular dementia.

**Table 5:** Associations of imaging variables with subcortical vascular dementia, *N* = 57.

	**Dementia**	
	**OR (95%CI)**	***p***
**MARS**		
Infratentorial	1.00 (0.93–1.07)	0.963
Deep	1.03 (0.99–1.08)	0.161
Lobar	1.00 (0.99–1.02)	0.739
Total	1.00 (0.99–1.01)	0.580
**ARWMC**		
Infratentorial	0.53 (0.10–2.80)	0.458
Basal ganglia	1.67 (0.74–3.78)	0.221
Subcortical	2.03 (1.24–3.32)	0.005^*^
Total	1.43 (1.09–1.89)	0.011^*^
**PVSE**		
Centrum semiovale	0.72 (0.36–1.42)	0.339
Basal ganglia	1.10 (0.44–2.74)	0.837
**Lesion quantity**		
ICH	1.00 (0.75–1.34)	1.000
Lacune	1.18 (1.02–1.35)	0.023^*^
**Lesion burden score**		
CAA-SVD score	2.33 (1.01–5.40)	0.047^*^
C1^†^	1.41 (1.09–1.83)	0.009^*^
C2^‡^	1.38 (1.08–1.76)	0.010^*^

*ICH, intracerebral hemorrhage; MARS, Microbleed anatomical rating scale; ARWMC, Age-related White Matter Change; PVSE, enlargement of the perivascular space; CAA, cerebral amyloid angiopathy; SVD, small vessel disease; OR, odd's ratio*.

*ORs determined by multivariate logistic regression, adjusted for age, sex, and CCI*;

* *p < 0.05*.

† *Sum of total MARS score and total ARWMC scale*.

‡ *Sum of total MARS score, total ARWMC scale, BG PVSE (≥20), and lacune (≥5)*.

## Discussions

This retrospective cohort study demonstrated that subcortical and total WMH burdens and the three composite scores were higher in patients with subcortical vascular dementia than in those without dementia. Although most regional measures of CMBs and WMHs correlated directly with one another, only subcortical and total WMH variables yielded desirable cognitive correlations. PVS ratings did not correlate with other imaging measures or any cognitive scores. Of the three composite scores, only C_1_ score correlated well with cognitive performance in terms of domain functioning. Of the imaging variables assessed, after adjusting for age, sex, and CCI, WMH load, lacune number, and all composite scores were associated with risk of subcortical vascular dementia. Collectively, these findings suggest that WMH load is more important in determining cognitive consequences than CMB burden and PVSE amount, even though accumulating imaging markers corresponding to CAA pathology (CAA-SVD score) or global SVD burden (C_1_ score and C_2_ score) contribute to dementia. Till date, this is the first study to examine the cognitive associations of comprehensive SVD imaging markers, regionally and totally as well as singularly and compositely, in the circumstance of advanced SVD of CAA and HA co-pathologies.

The enrolled subjects with multiple lobar CMBs had a > 80% prevalence of hypertension history, high CAA-SVD scores reflexive of heavy CAA pathological loads, and a > 80% prevalence of mixed CMBs, and consequently, advanced HA and CAA co-pathologies indeed existed in our cohort. We believe that the subjects included in our study belong to an intermediate between SVD type 3 and 4 in the SVD imaging spectrum (43). Although mixed CMBs are considered to be driven mostly by HA, both HA and CAA can cause cerebral hypoperfusion and enhance amyloid production (44), explaining the synergistic effects of HA (Hypertensive Angiopathy) and CAA on the cumulative changes in the vasculopathic and neurodegenerative processes in cognitive deterioration (8, 45).

Cerebral microbleeds and WMHs are known to be associated with each other in adults over 40 years old in the general population (46). In our cohort characterized by heavy CMB loads and a high prevalence of hypertension, CMB burden correlated strongly with WMH load, possibly supporting the notion that SVD might be a mediator of a complex relationship between CMBs and WMHs, both of which are well-established SVD manifestations. Vasculopathic changes implicated in both HA and CAA contexts make the relationship between CMBs and WMHs one of mutual promotion. These shared pathophysiologic characteristics of CMBs and WMHs provide putative mechanistic links.

In concordance with previous findings, lacunes were associated with deep CMBs (which are related to hypertension), but not with lobar CMBs (47). We found that ICH quantity correlates with non-lobar CMBs, rather than lobar CMBs. One previous study demonstrated that patients with mixed-location ICH/CMBs, regardless of the location of the symptomatic bleed, have relatively homogeneous clinical and radiologic profiles similar to patients with hypertensive ICH, rather than those with CAA-related ICH (48). This finding is probably attributable to a substantial presence of traditional vascular risk factors and evidence for hypertensive target-organ damage (48). Together with our results in a population with severe HA and CAA co-pathologies, a pronounced vascular risk factor burden may be the main driving force for microangiopathy as the major underlying SVD.

Enlargements of the perivascular space have been shown to be associated with other SVD markers, such as WMHs, lacunes, and CMBs, in discordance with our findings of PVSEs not associating with SVD-related markers (18, 49, 50). The lack of PVSE association could be partially explained by the high prevalence rates of severe CS PVSE (72.1%) and BG PVSE (62.3%) in our entire cohort (51).

Regarding the cognitive correlations of CMBs in our dementia group, deep and infratentorial CMBs correlated with short-term memory decline and visuoexecutive dysfunction, lobar CMBs with language impairment, and total CMBs with deficits in language and visuoexecutive function. CMB distributions greatly impact cognitive functions (52, 53). In previous studies, lobar CMBs correlated with impaired global cognition, attention, and visual executive function, BG CMBs with subnormal global cognition, and infratentorial CMBs with declined language ability and functional status (52–55). CMBs might reflect early neurodegenerative processes in a common pathogenesis of cognitive impairment, even though findings concerning effects of CMB location on cognition are still a matter of debate (56). Amyloid-related pathology and HA were shown to produce combined effects on the longitudinal progression of lobar CMBs and decline in attention and visuoexecutive function (13). As a result, it is possible that, in the context of multiple lobar CMBs (implying CAA), non-lobar CMBs (implying HA) may promote a synergism of CAA and HA in the development of cognitive impairment (13).

With respect to the cognitive associations of WMHs in our dementia group, subcortical, and total WMH burdens were significantly relevant to all cognitive domains, except memory registration and visuoexecutive function, while BG WMHs were related to global cognitive dysfunction and defective language, in line with numerous studies showing the associations of domain-specific cognitive profiles with WMH topographic changes (57–61). WMH distributions and loads are considered as predictors of cognitive impairment. Subcortical WMH scores in our study stood for the total WMH burdens in the frontal, parieto-occipital, temporal lobes, and regions adjacent to ventricle wall, which are believed to primarily interrupt both short and long connections to spatially distant brain areas. Therefore, subcortical WMHs can cause cognitive decline in multiple domains supported by the specific brain regions. In addition, cognitive dysfunction in vascular dementia may be the consequence of disconnection of the fronto-striatal circuits (62, 63). Cognitive tests involving language abilities were strongly related to damage to the fronto-subcortical connections, which are critical for both verbal abilities and global function (64–66). Our findings, along with previous literature, suggest that BG engages in global cognitive functioning contributing to performance in language tasks. We consider that WMHs considerably impact cognition and play a key role in determining the cognitive consequences.

No cognitive associations with regional PVSEs were identified in our dementia group. Advancing age and increasing burdens of CMBs and WMHs may obliterate the PVSE effect on cognition. Previous studies on the PVSE association with cognitive domain-specific analyses demonstrated discrepant findings: no PVSEs in any brain region associated with any cognitive domains, BG PVSEs associated with worse visuomotor speed, PVSEs in the hippocampus linked to better memory, and total PVSEs related to lower global cognition, non-verbal reasoning, and visuoexecutive function (18, 67–72). These inconsistent results imply insufficient evidence connecting either total or regional PVSEs with declines in specific cognitive domains.

The traditional global SVD index, derived by summing the aforementioned four established SVD MRI markers, can predict prognosis but is not feasible in our study (73). CAA-SVD, C_1_, and C_2_ scores were higher in our dementia group than in non-dementia group. Only C_1_ score significantly correlated with impairment in global cognition, recall memory, orientation, language, and visuoexecutive function as compared with CAA-SVD score (representative of entire CAA co-pathological burden) and C_2_ score (indicative of global SVD imaging burden). Unlike previous study showing an association of CAA-SVD score with cognitive functions, the discrepant finding may be attributed to varying population characteristics (74). Combining measures of total CMBs and WMHs (i.e., C_1_ score) added further correlation coefficients in the cognitive correlation analysis in contrast to regional and total imaging scores of CMBs or WMHs alone. Hence, we consider that incorporating parameters of CMBs and WMHs (i.e., C_1_ score), instead of global SVD imaging burden score (i.e., C_2_ score), is a potential imaging surrogate for domain-related cognitive impairment. Likewise, the entire CAA pathological burden score (i.e., CAA-SVD scoring) did not essentially correspond to domain-specific cognitive decline, specifically in a population with high burden of mixed pathologies in our study, which may undermine the cognitive associations of CAA-SVD scoring.

Of the imaging variables examined in this study, higher subcortical and total WMH loads, lacune number, CAA-SVD scores, C_1_ scores, and C_2_ scores posed increased risk of subcortical vascular dementia. CMBs, WMHs, and lacunes are well-described decisive imaging correlates of cognitive impairment and dementia of vascular and neurodegenerative processes (45, 75, 76). Accumulating imaging correspondences of CAA pathology (CAA-SVD score) and global SVD load (C_1_ score and C_2_ score) were consistently risky for dementia diagnosis. However, despite being a prevalent imaging finding in SVD, PVSEs have not expressed invariable predictability for cognitive decline. MARS scoring system that quantifies CMBs through numerical value, rather than on an ordinal scale, may account for no association of distributional CMB burdens with dementia risk in our study. Combining CMB and WMH markers (i.e., C_1_ score) reflective of two distinct underlying pathophysiological processes in CAA and HA did indeed show an incremental cognitive correlation as compared with CAA-SVD score and C_2_ score.

This study had several limitations. First, this study included a relatively small population from a single center. A larger cohort with longitudinal follow-up is necessary to clarify these findings and their reproducibility. Second, we did not use volumetric analysis and visual ratings may not precisely represent the entire lesion burden, but they are relatively easy to do and are readily applicable in clinical practice. Third, we did not analyze lobe-specific cognitive functions and distributions of individual imaging variables. Fourth, we did not analyze cortical microinfarcts, although some studies reported significant association between cognitive decline and cortical microbleeds. Finally, the cognitive associations of SVD imaging markers of the participants recruited from our stroke registry were not examined because comprehensive cognitive assessments were not routinely performed for these patients. This might account for some medium-sized but non-significant associations in our results.

In conclusion, individual imaging markers correlated differentially with one another and had differing relationships with cognitive domains. WMH burden seems to be the major imaging correlate of deficits across extensive cognitive domains. The present findings buttress previous research demonstrating HA and CAA co-pathologies contribute synergistically to the pathophysiological progression of cognitive decline in severe SVD.

## Data Availability Statement

The original contributions presented in the study are included in the article/supplementary material, further inquiries can be directed to the corresponding author/s.

## Ethics Statement

The studies involving human participants were reviewed and approved by Taichung Veterans General Hospital. Written informed consent for participation was not required for this study in accordance with the national legislation and the institutional requirements.

## Author Contributions

T-BC conceptualized and designed the study, analyzed the data, and did the critical revision of the manuscript. C-YL analyzed the imaging data, performed the statistical analysis, and wrote the manuscript. H-CC and S-RJ contributed to the acquisition and interpretation of the imaging data. T-BC, W-JL, and P-LC contributed to patient collection. J-PC performed the statistical analysis. All authors read and approved the submitted version.

## Funding

We are deeply indebted to Taichung Veterans General Hospital for providing the grants for this study (TCVGH-1103401A).

## Conflict of Interest

The authors declare that the research was conducted in the absence of any commercial or financial relationships that could be construed as a potential conflict of interest.

## Publisher's Note

All claims expressed in this article are solely those of the authors and do not necessarily represent those of their affiliated organizations, or those of the publisher, the editors and the reviewers. Any product that may be evaluated in this article, or claim that may be made by its manufacturer, is not guaranteed or endorsed by the publisher.
